# Comment on ‘Validation of a contemporary prostate cancer grading system using prostate cancer death as outcome’

**DOI:** 10.1038/bjc.2016.346

**Published:** 2016-10-25

**Authors:** Paolo Dell'Oglio, Armando Stabile, Giorgio Gandaglia, Alberto Briganti

**Affiliations:** 1 Department of Urology and Division of Experimental Oncology, URI, Urological Research Institute, IRCCS San Raffaele Scientific Institute, Milan, Italy; 2 Vita-Salute San Raffaele University, Milan, Italy

**Sir,**

We read with great interest the article by Berney and colleagues (Berney *et al*, 2016) who validated the new prostate cancer (PCa) grading system (Pierorazio *et al*, 2013) in a biopsy series of 988 conservatively treated PCa patients identified between 1990 and 2003. The authors (Berney *et al*, 2016) provided evidence that there is a strong correlation between the new PCa grading system and PCa death, relying on pre-treament models (Gleason score-GS at biopsy). Furthermore, they observed that the ‘worst’ GS has similar prognostic ability to the ‘overall’ GS in predicting PCa death. Berney and colleagues should be commended for their effort in validating the new PCa grading system using a stronger outcome, namely PCa mortality, compared with previous available studies (Epstein *et al*, 2015; Loeb *et al*, 2015; Spratt *et al*, 2016). Moreover, they tried to clarify a debated topic whether an ‘overall’ or ‘worst’ GS should be used in routine clinical practice. However, some points of the manuscript warrant discussion.

First, despite this being the first report that tested the new PCa grading system on the strongest available outcome, a discerning reader might argue that the findings of Berney and colleagues are fairly expected, given the fact that the GS is a known good predictor of PCa mortality (Bolton *et al*, 2015). In consequence, the authors do not provide any added advantage of the new PCa grading system that was not already known. The real unmet need that this report should have filled is: does the new PCa grading system improve the prediction of PCa death relative to the standard Gleason grading system? This comparison is mandatory to definitely prove the added value of the introduction of the new PCa grading system into daily clinical practice. Under this light, previous authors assessed the predictive ability of the new PCa grading system relative to the standard Gleason grading system on biochemical recurrence in patients treated with radical prostatectomy-RP (Epstein *et al*, 2015; Loeb *et al*, 2015; Spratt *et al*, 2016). For example, Epstein and colleagues (Epstein *et al*, 2015) were the first to validate the new PCa grading system in a multi-institutional series and to provide evidence that this new grading system is more accurate relative to the standard Gleason grading system. However, they were able to demonstrate only a limited increment in the predictive accuracy. Thereafter, Loeb and colleagues (Loeb *et al*, 2015) failed to observe higher predictive accuracy of the new PCa grading system relative to the standard Gleason grading system in a population-based setting.

Second, the accuracy of the new PCa grading system to predict PCa death was assessed exclusively relying on pretreatment models (i.e., GS at biopsy). Generally, GS at RP is more accurate to predict oncological outcomes relative to GS at biopsy, as proved by previous investigators that demonstrated higher predictive accuracy of the GS at RP relative to the GS at biopsy in predicting BCR (Epstein *et al*, 2015; Loeb *et al*, 2015).

Third, strict selection criteria were used to enroll patients in the current study, which might have biased its findings. For example, the investigators excluded patients older than 76 years. Currently, given the aging of the population and the increasing life expectancy worldwide (Tuljapurkar *et al*, 2000; United Nations, Department of Economic and Social Affairs, Population Division, 2015), the proportion of elderly is increasing. Consequently, it is mandatory to have evidence based that consider also elderly patients, which generally harboured worse disease characteristics (Shao *et al*, 2009; Dell’Oglio *et al*, 2016).

Fourth, the authors should be acclaimed to reassign GS of histological specimens from sextant biopsies performed between 1990 and 2003, according to a contemporary Gleason scoring system (Epstein, 2010). Certainly, this allowed to assess a hard outcome as PCa death, given the longer follow-up of non-contemporary patients. However, to date it should be more reasonable to evaluate less stronger outcome, namely clinical recurrence, relying on a cohort of patients that is treated with contemporary practice.

Last but not least, the authors did not assess the net benefit (Vickers and Elkin, 2006) of the new PCa grading system relative to the standard Gleason grading system for clinical decision-making, which is mandatory in a validation study. However, to the best of our knowledge, to date no studies overcome this issue.

In conclusion, the dilemma whether the new PCa grading system has higher predictive ability and superior clinical benefit relative to the standard Gleason grading system to predict PCa death, or it is only a user-friendly instrument to help patient counselling still persists. Future studies are needed to assess the discrimination and the net benefit of the new PCa grading system compared with the standard Gleason grading system on harder endpoint than BCR.
